# Metabolic Syndrome and Its Correlates Among Female Chronic Obstructive Pulmonary Disease Patients at a Rural Tertiary Health Care Center in Northern India

**DOI:** 10.7759/cureus.28611

**Published:** 2022-08-31

**Authors:** Ruchira Roy, Aditya K Gautam, Naresh P Singh, Adesh Kumar

**Affiliations:** 1 Department of Respiratory Medicine, Uttar Pradesh University of Medical Sciences, Etawah, IND; 2 Department of Community Medicine, Uttar Pradesh University of Medical Sciences, Etawah, IND

**Keywords:** ncep:atp iii, metabolic syndrome, hdl-c, gold, chronic obstructive pulmonary disease, bmi

## Abstract

Background: Chronic obstructive pulmonary disease (COPD) is a lung disease that is thought to result from chronic inflammation that may affect other organ systems. Similarly, metabolic syndrome includes central obesity, hypertriglyceridemia, low high-density lipoprotein cholesterol (HDL-C), hyperglycemia, and hypertension. The prevalence of metabolic syndrome and its associated factors among female COPD patients in northern India needs to be evaluated.

Aim and objectives: To find the prevalence of metabolic syndrome and its correlates among female chronic obstructive pulmonary disease patients at a rural tertiary health care center in northern India.

Materials and methods: A cross-sectional study was conducted between January 2019 and June 2020 at a rural tertiary health care center in northern India. The female patients who presented with symptoms of COPD and fulfilled the inclusion criteria were included and classified by Global Initiative for Chronic Obstructive Lung Disease (GOLD 2020) guideline while the clinical diagnosis of metabolic syndrome was made according to National Cholesterol Education Program: Adult Treatment Panel III (NCEP: ATP III) criteria.

Results: A total of 210 female COPD patients were included, the mean age of patients who had metabolic syndrome was 63.38±10.54 years. Metabolic syndrome was diagnosed in 60.48% of patients. There was a significant difference between female COPD patients with and without metabolic syndrome regarding body weight, BMI (body mass index), waist circumference, systolic blood pressure (SBP) and diastolic blood pressure (DBP), blood sugar, serum triglyceride, serum HDL-C. Whereas no significant difference was found between patients with and without metabolic syndrome group regarding smoking exposure, biomass fuel exposure, duration of biomass fuel exposure, mMRC (modified Medical Research Council) grading of breathlessness, GOLD grading of airflow limitation, route and duration of corticosteroid used. In our study, we also found a significant association between the severity of airflow limitation of COPD with the duration of biomass fuel exposure and BMI. Also, there was a significant association between biomass fuel exposure and the presence of cough in female COPD patients.

Conclusion: Metabolic syndrome is a prevalent entity in female COPD patients among the northern Indian population. Body weight, BMI, waist circumference, SBP, DBP, fasting blood sugar, serum triglyceride, and serum HDL-C have a significant impact on developing metabolic syndrome in female COPD patients. Duration of biomass fuel exposure and BMI also have a significant impact on the severity of airflow limitation in female COPD patients. So early detection and treatment of parameters of metabolic syndrome are important to reduce complications.

## Introduction

Chronic obstructive pulmonary disease (COPD) is a common, preventable, and treatable disease that is characterized by persistent respiratory symptoms and airflow limitation that is due to airway and/or alveolar abnormalities usually caused by significant exposure to noxious particles or gases (Global Initiative for Chronic Obstructive Lung Disease {GOLD} 2020). More than 3 million people died of COPD in 2012 accounting for 6% of all deaths globally [1]. A recent trend study using data from a general practice network demonstrated a constant rise in physician-diagnosed COPD in women [2]. The metabolic syndrome (syndrome X, insulin resistance syndrome) consists of a constellation of metabolic abnormalities that confer an increased risk of cardiovascular disease (CVD). The criteria for metabolic syndrome have evolved since the original definition by the World Health Organization in 1998, reflecting growing clinical evidence and analysis by a variety of consensus conferences and professional organizations. The major features of the metabolic syndrome include abdominal obesity, hypertriglyceridemia, low high-density lipoprotein cholesterol (HDL-C), hyperglycemia, and hypertension [3]. Several studies from different parts of the world have shown a higher prevalence of metabolic syndrome in COPD patients [4,5].

## Materials and methods

This was a hospital-based cross-sectional study carried out at a rural tertiary care center in northern India, conducted between January 2019 and June 2020. We enrolled 210 female COPD patients of age 40 years and above, who attended the outdoor and indoor patient departments at the study place. Inclusion criteria were those female COPD patients who were aged 40 years and above, who were hemodynamically stable, co-operative, and who have provided consent to participate in the study. Whereas female COPD patients with recent myocardial infarction, respiratory failure, pregnancy, multiorgan failure, and all other patients visiting the study place with other diseases were excluded from the study. Data was collected by the self-reported method of data collection. Tools used for data collection included a semi-structured, pre-designed questionnaire with sections on socio-demographic profile (age, occupational status, socio-economic status, education, smoking, and other addiction habits), a clinical profile sheet which had details of diagnosis of the patient, pulmonary function test (PFT), spirometric grading of airflow limitation of COPD (GOLD 1-4) [1], grading of dyspnoea according to Modified Medical Research Council (mMRC) dyspnoea scale [1], duration of illness, number of exacerbations, history of smoking (cigarette/bidi/hukka), bio-mass fuel exposure history, treatment history, blood pressure measurement, anthropometric measurements (height, weight, BMI, waist circumference), lipid profile (serum triglyceride, serum high-density lipoprotein cholesterol), fasting blood sugar measurement. COPD was diagnosed according to guidelines adopted and recommended by GOLD 2020 [1]. All patients enrolled in the study were subjected to chest X-ray postero-anterior (PA) view and spirometry to confirm the diagnosis and also to exclude other pathology of the chest.

Body weight and height were measured and the BMI (body mass index) was calculated by dividing the weight in kilograms by the height in meters squared (kg/m^2^), which was classified as underweight (<18.5 kg/m^2^), normal weight (18.5-24.9 kg/m^2^), overweight (25-29.9 kg/m^2^) and obese (>30 kg/m^2^) [6]. The blood pressure was measured according to the American Heart Association’s recommendations. Blood pressure measurements were obtained from both arms in the supine position after a 15-minute resting period and the highest measurement was recorded and used for analysis [6]. The National Cholesterol Education Program: Adult Treatment Panel III (NCEP: ATP III) criteria were used in the diagnosis of metabolic syndrome (Table 1).

**Table 1:** Criteria for clinical diagnosis of metabolic syndrome according to National Cholesterol Education Program: Adult Treatment Panel III (NCEP: ATP III) cm:centimeter, mg/dL: milligram/decilitre, mmol/L: millimole/litre, mmHg: millimeter of mercury, HDL-C: High-density lipoprotein cholesterol

Measure (any 3 of 5 constitute for diagnosis of metabolic syndrome)	Categorical cut points
Elevated waist circumference	≥102 cm (≥40 inches) in men & ≥88 cm (≥35 inches) in women
Elevated triglycerides	≥150 mg/dL (1.7 mmol/L) or,on drug treatment for elevated triglycerides
Reduced HDL-C	<40 mg/dL (1.03 mmol/L) in men & <50 mg/dL (1.3 mmol/L) in women or, on drug treatment for reduced HDL-C
Elevated blood pressure	≥130 mmHg systolic blood pressure or,≥85 mmHg diastolic blood pressure or, on antihypertensive drug treatment in a patient with a history of hypertension
Elevated fasting glucose	≥100mg/dL or,on drug treatment for elevated glucose

If the participants were using antihypertensive or antidiabetic drugs, they were considered to be having high blood pressure or high fasting glucose [3]. Written consent was taken from all the cases. Ethical committee clearance was taken from the institution. Ethical Committee of Uttar Pradesh University of Medical Sciences (UPUMS) issued approval vide letter number 1309/UPUMS/Dean(M)/Ethical/2020-21, dated October 13, 2020. The ethical clearance number is 170/2018.

To measure waist circumference, the top of the right iliac crest was located. A measuring tape in a horizontal plane around the abdomen at the level of mid-point of the iliac crest and lower border of the lowest rib was placed. Before reading a tape measure, it was ensured that the tape is snug but does not compress the skin and is parallel to the floor. Measurement was made at the end of a normal expiration. Lower waist circumference cut point e.g. 90 cm (35 inches) in men and 80 cm (31 inches) in women is appropriate for Asian Americans [3]. Fibrates and nicotinic acid were the most commonly used drugs for elevated triglyceride (TG) and reduced HDL-C. Patients taking one of these drugs were presumed to have high triglyceride and low HDL-C [3].

Data analysis

The data thus collected was entered into a Microsoft office excel worksheet and the result was analyzed using SPSS software version 25 (IBM Corp., Armonk, NY) and appropriate statistical interpretation was done using proportions, mean, standard deviation, chi-square test, and students’ unpaired t-test.

## Results

A total of 210 female COPD patients were included in the study with a mean±SD age of 63.38±10.54 years with a range of 40 to 95 years (Table 2).

**Table 2:** Central tendency measurement of age and anthropometric variables of study subjects Kgs: Kilograms, BMI: Body mass index, Kg/m^2^: Kilogram/meter^2^

Variables	Mean	Median	Standard deviation	Range	Minimum	Maximum
Age (in years)	63.38	62.00	10.543	55	40	95
Body weight (in kgs)	52.119	50.00	10.876	47.0	33.0	80.0
Height (in meters)	1.535	1.54	0.027	0.18	1.42	1.60
BMI (kg/m^2^)	22.15	21.46	4.614	19.88	14.74	34.62

**Figure 1:**
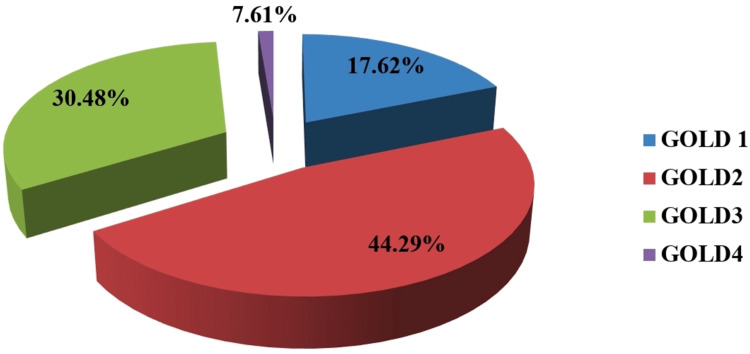
Pie chart showing the frequency distribution of study subjects according to the severity of airflow limitation (GOLD grading) GOLD: Global Initiative for Chronic Obstructive Lung Disease

Out of 210 female COPD patients, a maximum i.e. 93 (44.29%) patients were diagnosed in GOLD 2, 64 (30.48%) were in GOLD 3, and the least i.e., 16 (7.61%) patients were in GOLD 4 (Figure 1). 

**Figure 2:**
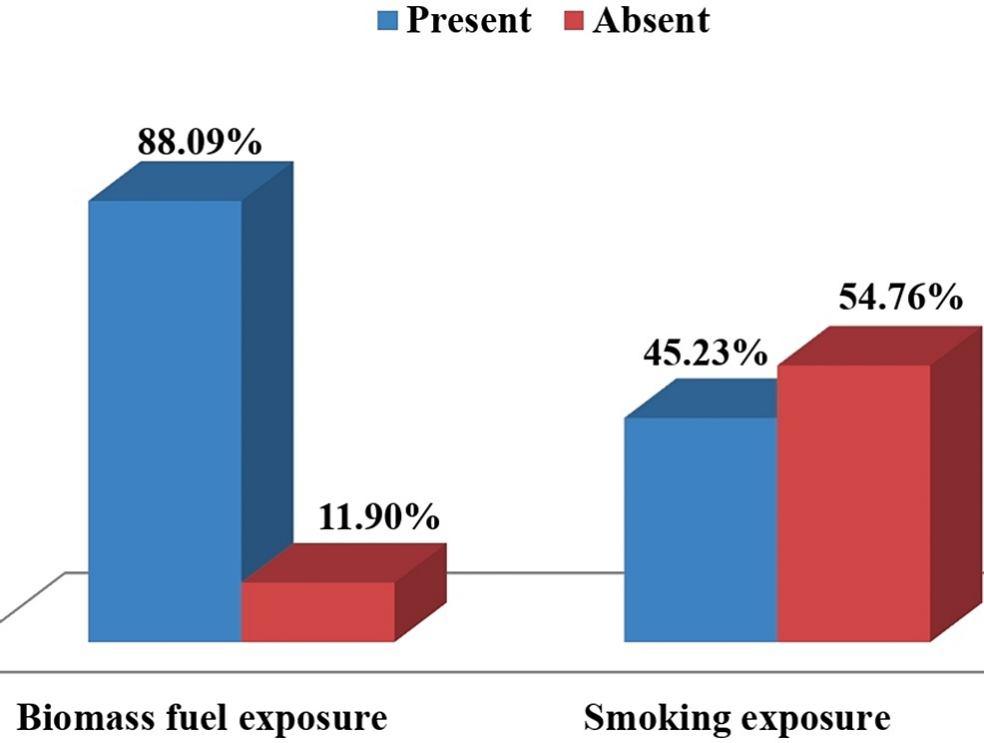
Group bar graph showing the distribution of study participants according to biomass fuel and smoking exposure

In our study, out of 210 female COPD patients, 88.09% were exposed to biomass fuel and 11.90% were not exposed to biomass fuel, also 45.23% were smokers and 54.76% were non-smokers (Figure 2). 

**Figure 3:**
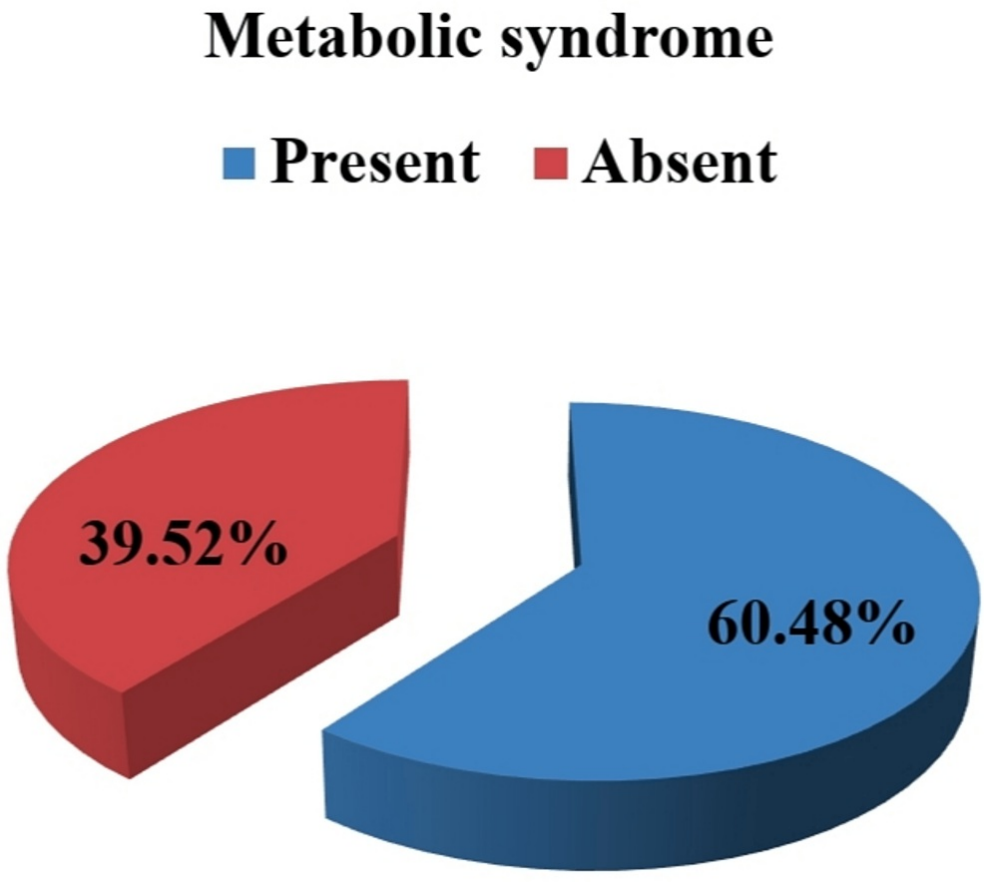
Distribution of metabolic syndrome in study subjects according to NCEP: ATP III criteria NCEP: ATP III criteria: National Cholesterol Education Program: Adult Treatment Panel III criteria

In our study, the prevalence of metabolic syndrome among study subjects was 60.48% according to NCEP: ATP III criteria (Figure 3). 

In this study, a comparison between female COPD patients with and without metabolic syndrome was done regarding mean±SD values of various parameters. There was a statistically significant difference found between the two groups regarding body weight, BMI, waist circumference, systolic blood pressure (SBP), diastolic blood pressure (DBP), fasting blood sugar, serum triglyceride, and serum HDL-C (p-value <0.05) (Table 3). 

**Table 3:** Comparison of various parameters with the presence of metabolic syndrome in study subjects Kgs: Kilograms, BMI: Body mass index, Kg/m^2^: Kilogram/meter^2^, cms: Centimeters, SBP: Systolic blood pressure, DBP: Diastolic blood pressure, mmHg: Millimeter of mercury, mg/dL: Milligram/decilitre

Variables (Mean ± SD)	Metabolic Syndrome		Statistical interpretation (Students’ Unpaired t-test)
	Present	Absent	
Age (in years)	64.1±10.4	62.3±10.8	P=0.22
Body weight (in kgs)	55.9±10.7	46.4±8.3	P<0.001
BMI (kg/m^2^)	23.8±4.5	19.6±3.4	P<0.001
Waist Circumference (cms)	84.2±8.9	75.7±6.6	P<0.001
SBP (mmHg)	129.3±18.0	124.0±11.9	P=0.012
DBP (mmHg)	86.3±9.7	79.3±9.7	P<0.001
Fasting blood sugar (mg/dL)	114.2±36.4	94.9±28.8	P<0.001
Serum triglyceride (mg/dL)	151.1±51.3	110.2±34.2	P<0.001
Serum HDL-C (mg/dL)	53.9±18.1	61.4±10.4	P=0.003

In our study, we also compared the female COPD patients with and without metabolic syndrome regarding smoking exposure, biomass fuel exposure, duration of biomass fuel exposure, mMRC grading of breathlessness, GOLD airflow limitation grading, route and duration of corticosteroid used and found no statistically significant difference between these two groups (Table 4).

**Table 4:** Association between various clinical parameters versus the presence of metabolic syndrome

Variables	Status	Metabolic syndrome		Total n (%)	Statistical interpretation (Chi-square test)
		Present n (%)	Absent n (%)		
Smoking exposure	Present	58 (61.1)	37 (38.9)	95 (45.24)	P=0.88
	Absent	69 (60.0)	46 (40.0)	115 (54.76)	
Biomass fuel exposure	Present	110 (59.5)	75 (40.5)	185 (88.1)	P= 0.41
	Absent	17 (68.0)	8 (32.0)	25 (11.90)	
Duration of exposure of biomass fuel	Not exposed	17 (70.8)	7 (29.2)	24 (11.43)	P=0.39
	≤10 years	10 (62.5)	6 (37.5)	92 (43.81)	
	11-20 years	54 (58.7)	38 (41.3)	16 (7.62)	
	21-40 years	37 (55.2)	30 (44.8)	67 (31.90)	
	> 40 years	9 (81.8)	2 (18.2)	11 (5.24)	
Modified Medical Research Council dyspnoea grading	No Breathlessness	6 (66.7)	3 (33.3)	9 (4.29)	P=0.75
	Grade0	27 (69.2)	12 (30.8)	39 (18.57)	
	Grade1	37 (56.9)	28 (43.1)	65 (30.95)	
	Grade2	32 (60.4)	21 (39.6)	53 (25.24)	
	Grade3	21 (60.0)	14 (40.0)	35 (16.67)	
	Grade4	4 (44.4)	5 (55.6)	9 (4.29)	
GOLD airflow limitation grading	GOLD 1	24 (64.9)	13 (35.1)	37 (17.62)	P= 0.84
	GOLD 2	57 (61.3)	36 (38.7)	93 (44.29)	
	GOLD 3	36 (56.3)	28 (43.7)	64 (30.48)	
	GOLD 4	10 (62.5)	6 (37.5)	16 (7.61)	
Route of corticosteroid use	Not used	19 (51.4)	18 (48.6)	37 (17.62)	P=0.36
	Oral	39 (59.1)	27 (40.9)	66 (31.43)	
	Inhaled	69 (64.5)	38 (35.5)	107 (50.95)	
Duration of corticosteroid use	Not used	19 (51.4)	18 (48.6)	37 (17.62)	P=0.44
	Less than 1year	64 (61.5)	40 (38.5)	104 (49.52)	
	More than 1year	44 (63.8)	25 (36.2)	69 (32.86)	

In the present study, there was a significant association found between the duration of biomass fuel exposure and BMI with various grades of GOLD airflow limitation (p-value <0.001) (Table 5).

**Table 5:** Association of the severity of airflow limitation of COPD (GOLD grading) with the duration of biomass fuel exposure and BMI status COPD: Chronic obstructive pulmonary disease, GOLD: Global Initiative for Chronic Obstructive Lung Disease

Variables	Status	GOLD airflow limitation grading				Total (n%)	Statistical Interpretation (Chi-square test)
		GOLD 1 (n%)	GOLD 2 (n%)	GOLD 3 (n%)	GOLD 4 (n%)		
Duration of exposure of biomass fuel	Not exposed	15(40.5%)	8(8.6%)	0(0.0%)	1(6.3%)	24(11.42)	P<0.001
	<10years	3(35.1%)	8(8.6%)	5(7.8%)	0(0.0%)	16 (7.61)	
	10-20years	13(35.1%)	44(47.3%)	31(48.4%)	4(25.0%)	92(43.80)	
	21-40years	6(16.2%)	26(28.0%)	24(37.5%)	11(68.8%)	67(31.90)	
	>40years	0(0.0%)	7(7.5%)	4(6.3%)	0(0.0%)	11 (5.23)	
Categories of BMI	Underweight	3(8.1%)	20(21.5%)	15(23.4%)	6(37.5%)	44(20.95)	P<0.001
	Normal weight	18(48.6%)	49(52.7%)	33(51.6%)	4(25.0%)	104(49.52)	
	Overweight	16(43.2%)	21(22.6%)	16(25.0%)	4(25.0%)	57 (27.14)	
	Obese	0(0.0%)	3(3.2%)	0(0.0%)	2(12.5%)	5 (2.38)	

In this study, we also found that there was a significant association present between biomass fuel exposure and the presence of cough (p<0.05), but no significant association was found between smoking and cough status (p=0.46) (Table 6).

**Table 6:** Association of the presence of cough with biomass fuel and smoking exposure

Variables	Status	Cough status		Total n (%)	Statistical interpretation (Chi-square test)
		Present n (%)	Absent n (%)		
Biomass fuel exposure	Present	166 (89.7)	19 (10.3)	185(88.09)	P=0.012
	Absent	18 (72.0)	7 (28.0)	25(11.90)	
Smoking exposure	Present	85 (89.5)	10 (10.5)	95(45.23)	P=0.46
	Absent	99 (86.1)	16 (13.9)	115(54.76)	

## Discussion

In this study mean±SD age of the female COPD patients was 63.38±10.54 years, which is almost similar to previous studies which reported (66.66±8.72 years) [7] and (62.69±0.84 years) [8]. In our study, we could not find any difference between COPD patients with and without metabolic syndrome group regarding age (p-value=0.22). This result was in agreement with the result of a study done in Spain [5], which also could not find any significant association between age and the presence of metabolic syndrome (p-value=0.94).

Patients with mild obstructive defect, that is forced expiratory volume (FEV1)≥80% of predicted are usually in the pre-symptomatic stage and are not likely to come to medical attention unless they develop an exacerbation or lower respiratory tract infection. Whereas maximum patients who were in GOLD 4, i.e. patients with very severe airflow limitation were excluded due to associated hemodynamic instability and other exclusion criteria. So in our study, we could include less number of patients of GOLD 1 and 4. This accounts for the fact that the maximum number i.e. 96.5% of female COPD patients with metabolic syndrome were in GOLD 2 and 3. This result was in concurrence with the study result which was done in Karnataka [9], as they also found that maximum COPD patients (73.22%) with metabolic syndrome were in GOLD 2 and 3, i.e., moderate and severe airflow limitation, respectively.

In the present study, we could not show any significant difference between female COPD patients with and without metabolic syndrome group regarding the severity of airflow limitation according to GOLD grading, which was similar to a study done by Watz et al. [4], who have reported that half of the patients included in their study had metabolic syndrome irrespective of disease stage and severity.

Similarly, in the present study, there was no significant difference between metabolic syndrome and without metabolic syndrome group of female COPD patients regarding mMRC grading of breathlessness which can be explained by the fact that COPD has a higher negative impact on quality of life than metabolic syndrome (physical limitation due to shortness of breath), which is ameliorating the effect in patients having both diseases [10].

In agreement with the study done in Spain [5], we also could not find any significant association between smoking status and the presence of metabolic syndrome. This result can be explained by the fact that smoke is the biggest factor in developing COPD and the effect of developing metabolic syndrome can be reduced. Moreover, nicotine may be an appetite suppressant and lower weight thus decreasing the prevalence of metabolic syndrome [11].

There was no significant association between the presence and duration of biomass fuel exposure and the presence of metabolic syndrome in female COPD patients in the present study, but more studies are still needed in this regard. Whereas significant association was found between the duration of biomass fuel exposure and severity of airflow limitation in COPD patients, which was not in agreement with the study result of Halbert et al. [12]. This may be because of the difference in type and duration of biomass fuel exposure or the difference in severity pattern of study subjects in their study. In our study, we found that 54.76% of study subjects were non-smokers, which was almost similar to the study result of Halbert et al. [12], who also found that 41.70% of study subjects were non-smokers.

Prevalence of metabolic syndrome according to NCEP: ATP III criteria in female COPD patients was found to be 60.48%. Researchers of Karnataka, India, [5] have found the prevalence of metabolic syndrome in COPD patients was more frequent in female patients (59.5%) than in male patients (40.8%) and the percentage of the female COPD patient was almost similar to our study.

A significant difference was found between the metabolic and non-metabolic syndrome group of female COPD patients regarding BMI (p-value<0.001), which was in concurrence with the study result done in the Netherlands [13], which also found a significant difference.

In the present study, a significant association was present between BMI and severity of airflow limitation according to GOLD grading and this was seen that in all categories of BMI, maximum patients were found in GOLD 2, which can be explained by the fact that the weight loss, muscle wasting and loss of fat-free mass is more prominent in late stages in COPD also known as Obesity Paradox [14].

In our study we also found a significant difference between female COPD patients with and without metabolic syndrome group regarding body weight, waist circumference, SBP, DBP, fasting blood sugar, serum triglyceride level, and serum HDL-C level, which was almost similar to the study done in Spain [5], as they also found significant difference regarding SBP, fasting blood sugar, serum triglyceride level, serum HDL-C level between the two groups. 

The pathological mechanisms responsible for hypertension in COPD are hypoxia-related vasoconstriction, free radical injury, endothelial dysfunction, and arterial stiffness [15-17]. A study concluded that reduced lung function is an important risk factor for the development of diabetes in COPD [18]. The association of COPD with diabetes is being increasingly recognized. It is demonstrated that approximately 3% to 12% of subjects with COPD had diabetes [19].

Most of the studies showed an inconclusive pattern of dyslipidemia in the COPD population. In the present study, findings of dyslipidemia i.e. elevated TG and decreased HDL levels were similar to many previous studies [20,21]. In contrast to our study, many studies have revealed elevated HDL-C [22-24]. This can be explained by the difference in lifestyle and socioeconomic status of the other populations.

A study done in Spain [5] has shown significantly higher use of inhaled corticosteroids in COPD patients who had metabolic syndrome in comparison to COPD patients without metabolic syndrome, which was on contrary to our study result, which could not show any significant difference between COPD patients with and without metabolic syndrome group regarding route and duration of corticosteroid used.

In the present study, we also found a significant association between the presence of cough and biomass fuel exposure but there was no association found between smoking exposure and the presence of cough. This may be explained as less number of female patients are usually exposed to smoking but more exposed to biomass fuel in our study population.

Study limitations

There are some limitations in the present study. The result of this study cannot be generalized as this is a hospital-based study. There was no control group in our study, so we could not assess the role of COPD in the pathogenesis of metabolic syndrome. This was a cross-sectional study, so we could not establish causal relationships with clinical outcomes. In our study the sample size is small. A study with a larger sample size with a longer duration will be required to get a better outcome.

## Conclusions

From our study, we can conclude that metabolic syndrome is commonly present in female COPD patients in northern India. There was a significant impact of body weight, BMI, waist circumference, SBP, DBP, fasting blood sugar, serum triglyceride, and serum HDL-C levels on the presence of metabolic syndrome in female COPD patients. Also, we can see the significant associations between the severity of COPD with the duration of biomass fuel exposure and with the BMI of the patients. Biomass fuel exposure has a significant impact on the presence of cough in female COPD patients, but smoking exposure, biomass fuel exposure, duration of biomass fuel exposure, mMRC grading of breathlessness, GOLD severity of airflow limitation, route and duration of corticosteroid used had no significant impact on the presence of metabolic syndrome in female COPD patients in our study. So early detection and treatment of parameters of metabolic syndrome are important to reduce complications.
